# Anti-biofilm effectiveness of protocols for cleaning complete dentures in hospitalized patients: a randomized controlled trial

**DOI:** 10.1590/1678-7757-2022-0381

**Published:** 2024-02-26

**Authors:** Anna Clara Gurgel GOMES, Janaina Gomes MACIEL, Amanda Aparecida Maia Neves GARCIA, Letycia Accioly Simões COELHO, Giulia Murcia RODRIGUES, Vinicius Carvalho PORTO, Grigorios POLYZOIS, Marlise Inêz KLEIN, Simone SOARES, Vanessa Migliorini URBAN, Karin Hermana NEPPELENBROEK

**Affiliations:** 1 Universidade de São Paulo Faculdade de Odontologia de Bauru Departamento de Prótese e Periodontia Bauru SP Brasil Universidade de São Paulo, Faculdade de Odontologia de Bauru, Departamento de Prótese e Periodontia, Bauru, SP, Brasil.; 2 Universidade de São Paulo Faculdade de Odontologia de Bauru Departamento de Dentística, Endodontia e Materiais Odontológicos Bauru SP Brasil Universidade de São Paulo, Faculdade de Odontologia de Bauru, Departamento de Dentística, Endodontia e Materiais Odontológicos, Bauru, SP, Brasil.; 3 National and Kapodistrian University of Athens School of Dentistry Athens Greece National and Kapodistrian University of Athens, School of Dentistry, Athens, Greece.; 4 Universidade Estadual de Campinas Faculdade de Odontologia de Piracicaba Departamento de Diagnóstico Oral Piracicaba SP Brasil Universidade Estadual de Campinas, Faculdade de Odontologia de Piracicaba, Departamento de Diagnóstico Oral, Piracicaba, SP, Brasil.; 5 Universidade Estadual de Ponta Grossa Departamento de Odontologia Ponta Grossa PR Brasil Universidade Estadual de Ponta Grossa, Departamento de Odontologia, Ponta Grossa, PR, Brasil.

**Keywords:** Denture, Biofilms, Denture Cleansers, Hospital Care

## Abstract

Denture biofilm acts as a potential reservoir for respiratory pathogens, considerably increasing the risk of lung infections, specifically aspiration pneumonia, mainly 48h after hospital admission. The establishment of a straightforward, affordable, and applicable hygiene protocol in a hospital environment for the effective control of denture biofilm can be particularly useful to prevent respiratory infections or reduce the course of established lung disease.

**Objectives:**

To evaluate the anti-biofilm effectiveness of denture cleaning protocols in hospitalized patients.

**Methodology:**

The maxillary complete dentures (MCDs) of 340 hospitalized participants were randomly cleaned once using one of the following 17 protocols (n=20): brushing with distilled water, toothpaste, or neutral liquid soap (controls); immersion in chemical solutions (1% sodium hypochlorite, alkaline peroxide, 0.12% or 2% chlorhexidine digluconate), or microwave irradiation (650 W for 3 min) combined or not with brushing. Before and after the application of the protocols, the biofilm of the intaglio surface of the MCDs was evaluated using two methods: denture biofilm coverage area (%) and microbiological quantitative cultures on blood agar and Sabouraud Dextrose Agar (CFU/mL). Data were subjected to the Wilcoxon and Kruskal-Wallis tests (α=0.05).

**Results:**

All 17 protocols significantly reduced the percentage area of denture biofilm and microbial and fungal load (*P*<0.05). The highest percentage reductions in the area of denture biofilm were observed for 1% hypochlorite solution with or without brushing and for 2% chlorhexidine solution and microwave irradiation only in association with brushing (*P*<0.05). The greatest reductions in microbial and fungal load were found for the groups that used solutions of 2% chlorhexidine and 1% hypochlorite and microwave irradiation, regardless of the association with brushing (P<0.05).

**Conclusions:**

A single immersion for 10 min in 1% sodium hypochlorite, even in the absence of brushing, proved to be a straightforward, rapid, low-cost, and effective protocol for cleaning the dentures of hospitalized patients.

## Introduction

The leading cause of pneumonia in elderly patients is related to the aspiration of food and oral microorganisms and reflux. Its treatment and especially its prevention are essential.^1^ Compared with non-aspiration pneumonia, aspiration pneumonia (AP) results in more admissions to intensive care units (ICU), the need for mechanical ventilation, the increase in the length of stay, and a mortality rate reaching 76% in people over 75 years of age.^1^AP is estimated to represent from 5 to 24% of community-acquired pneumonia. Still, incidence of the disease acquired in hospitals (nosocomial) varies from 29.7 to 70% and is 8 to 10 times higher in individuals over 70 years of age; the second highest occurrence of hospital infections.^2^

Current data suggest that poor oral health is the major risk factor for AP in older people.^3^ One in 10 deaths could be avoided in this population by adequate and regular oral hygiene. The microbial composition of dental biofilm highlights species of microorganisms related to general infections, with a clear relation between oral and systemic comorbidities.^3^ This association was also observed with denture biofilm, especially for respiratory tract infections, particularly in relation to AP. Similar to dental biofilm, denture biofilm contains respiratory pathogens, considered potential triggers of AP, especially in poor hygiene.^4,5^

In this context, users of removable dentures, mostly elderly individuals, deserve special attention^4,5^ since it was found that wearing these prostheses, especially during sleep,^6^ is a risk predictor for the incidence of pneumonia in the geriatric population.^7^Even greater attention should be directed to hospitalized elderly people because the presence of oral and denture biofilm associated with compromised host immunity and the aspiration of the contents present in the oropharynx into the lower respiratory tract increases the risk of AP, especially 48 h after hospitalization.^8^ Furthermore, the greater lack of autonomy also leads to less satisfactory cleaning of dentures during hospitalization. Another critical factor is that medical and nursing teams lack knowledge about oral health and hygiene care with dentures due to the absence of educational programs and/or the lack of a hospital dental team.^9^

The importance of oral hygiene in hospitalized patients was demonstrated with a reduction of up to 40% in cases of pneumonia after tooth brushing and rinsing with chlorhexidine digluconate.^8^ Despite these favorable data obtained with the removal of dental biofilm, information on cleaning protocols for removable dentures in hospitalized patients is lacking, and most users still clean their dentures by simply rinsing them in water or brushing them with toothpaste, soap or even water because it is easy, simple, and inexpensive.^10^However, when used alone, brushing has poor effectiveness in removing denture biofilm,^11-16^ especially when performed by older individuals with compromised manual dexterity and visual acuity.^10^To overcome these limitations, brushing should be combined with microwave irradiation or immersion in chemical cleaning agents.^14,17-27^These protocols are ideally recommended as methods of denture disinfection in order to effectively and completely remove biofilm after one application.^26^Unlike home denture hygiene practices to control biofilm, which are used daily by denture wearers,^28^such cleaning protocols are recommended for short periods of time^23^, including the patient’s stay in a hospital. Denture cleaning protocols using solutions of 1% sodium hypochlorite, 2% chlorhexidine digluconate and alkaline peroxide, as well as microwave irradiation at 650 W demonstrated *in vivo* and *in vitro* antimicrobial effectiveness on denture base materials, even after a single application.^14,17-27^ Furthermore, such protocols, after successive application cycles, are unable to adversely affect the physical and mechanical properties of denture base materials^17,29-31^ and acrylic artificial teeth.^32-34^

Considering that denture biofilm may be a reservoir for respiratory pathogens, straightforward, efficient, affordable, and applicable protocols for cleaning dentures should be established in hospital environments in order to prevent respiratory infections, especially AP, as well as to mitigate the course of established lung diseases.^35^ Hence, this randomized clinical trial (RCT) was designed to compare different methods of cleaning the dentures of hospitalized patients to establish an effective protocol for removing denture biofilm. The tested hypothesis was that the reduction of the denture biofilm coverage area and the microbial and fungal load would vary according to the denture cleaning protocol.

## Methodology

This study was a blind, parallel-arm RCT (Registration number in the Brazilian Clinical Trials Registry - RBR-7gstxs6; March 25, 2022), approved by the institutional Ethics Committee (C.A.A.E – 48753215.3.0000.5417). Individuals admitted to Hospital Beneficência Portuguesa and Hospital de Base, Bauru, Brazil, were recruited from October 2018 to November 2021. Inclusion criteria were participants wearing a maxillary complete denture (MCD). Participants with fractured and/or relined MCDs were excluded from this study.

After written consent had been obtained, the medical records of the selected participants were investigated regarding the general risk factors involved with microbial colonization of MCD and, therefore, with denture biofilm: age, sex, diagnostic hypothesis, hospitalization time, and use of antibiotics, antifungals, and steroids.^16^ For antibiotics and antifungals, the type (salt name) and day of treatment when the biofilm was collected for this study were recorded. Moreover, two local risk factors related to the density of the denture biofilm and the prevalence of oral infections were considered in this RCT: denture age and nocturnal denture wear.^6,16^

The stratified block randomization of the samples was done by the open Epi random software (https://www.openepi.com/Random/Random.htm) to create similar groups of participants with baseline characteristics that could influence prognosis other than those tested (general and local risk factors). Participants (N=359) were randomly allocated to one of the 17 study groups according to the denture cleaning protocol, that is, three control and 14 experimental groups. Each protocol was performed once. In the control groups, MCD were brushed with a new soft brush (Colgate Clean, São Bernardo do Campo, SP, Brazil) and distilled water, neutral liquid hand soap (Lifebuoy Original, Bolton, Lancashire, England), or toothpaste (Colgate Total^®^ 12, Colgate-Palmolive Industrial Ltda., São Bernardo do Campo, SP, Brazil). Experimental groups used cleaning solutions of 1% sodium hypochlorite, 0.12% and 2% chlorhexidine digluconate (Rioquimica Indústria Farmacêutica S/A, São José do Rio Preto, SP, Brazil), alkaline peroxide tablets (Corega Tabs, GlaxoSmithKline Brasil Ltda., Rio de Janeiro, RJ, Brazil), or microwave irradiation (Model Sensor Crisp 38, Brastemp, Double Emission System, Manaus, AM, Brazil) with or without prior brushing of the denture. It is worth mentioning that, in addition to the 2% chlorhexidine solution, which is considered effective for disinfecting dentures,^19-22,25,26^cleaning protocols included the same solution in a concentration of 0.12% since this mouthrinse is generally available in hospitals, as was the case in the two participants in this trial. Participants were randomly assigned to one of the following study groups (with at least 20 individuals each):

**BRU/DW**: Brushing with sterile distilled water for 2 min;^12,13,36^

**BRU/TP**: Brushing with toothpaste for 2 min;^37^

**BRU/SP**: Brushing with 5 mL of neutral liquid hand soap for 2 min;^38^

**CHX-0.12**: Immersion in 150 mL of 0.12% chlorhexidine digluconate for 10 min;^20^

**BRU+CHX-0.12**: Brushing with distilled water for 2 min,^12,13,36^followed by immersion in 150 mL of 0.12% chlorhexidine digluconate for 10 min;^20^

**CHX-2**: Immersion in 150 mL of 2% chlorhexidine digluconate for 10 min;^17,18^

**BRU/CHX-2**: Brushing with 100 mL of 2% chlorhexidine digluconate for 90 s;^24^

**BRU+CHX-2**: Brushing with distilled water for 2 min,^12,13,36^followed by immersion in 150 mL of 2% chlorhexidine digluconate for 10 min;^18^

**HYP**: Immersion in 150 mL of 1% sodium hypochlorite for 10 min;^17,19^

**BRU+HYP**: Brushing with distilled water for 2 min,^12,13,36^followed by immersion in 150 mL of 1% sodium hypochlorite for 10 min;^17,19^

**BRU/HYP**: Brushing with 100 mL of 1% sodium hypochlorite for 90 s;^24^

**PER1**: Immersion in 150 mL of solution prepared with 1 alkaline peroxide tablet and warm water (37ºC) for 5 min;^13,14^

**BRU+PER1**: Brushing with water for 2 min,^12,13,36^followed by immersion in 150 mL of 1 tablet of alkaline peroxide solution for 5 min (37°C);^13,14^

**PER2**: Immersion in 150 mL of solution prepared with 2 tablets of alkaline peroxide and warm water (37ºC) for 5 min;^21^

**BRU + PER2**: Brushing with water for 2 min,^12,13,36^followed by immersion in 150 mL of 2 tablets of alkaline peroxide solution for 5 min (37ºC);^21^

**MW**: Immersion in 200 mL of sterile distilled water for microwave irradiation for 3 min at 650 W;^15,26^

**BRU+MW**: Brushing with distilled water for 2 min,^12,13,36^followed by immersion in 200 mL of sterile distilled water for microwave irradiation for 3 min at 650 W.^15,26^

Before the application of the cleaning protocols, all toothbrushes were sterilized in a microwave at 650 W for 6 min.^39^ At the end of each protocol, the denture was immersed in 200 mL of sterile distilled water for 3 min for complete rinsing.^23^ In the presence of remaining teeth or other prostheses in the mandibular arch, the patient or their guardian received oral hygiene instructions from the responsible researcher, as well as a toothbrush and toothpaste. Furthermore, in the detection of oral lesions (e.g., denture stomatitis, fibrous hyperplasia, median rhomboid glossitis, etc.) or dental diseases such as caries and periodontal disease, the participant was duly referred to the clinic at the Bauru School of Dentistry, University of São Paulo, Bauru, Brazil.

Participants were unaware of the denture cleaning protocol. Even participants who shared the same hospital room were unaware of the interventions, which were always and individually performed by one trained clinician in a separate outpatient room to prevent the contamination of the study. Due to the nature of the intervention, blinding the operator to patient allocation was impossible.

To investigate the effectiveness of cleaning protocols, the primary outcomes of this study were the denture biofilm coverage area and the number of viable microbial cells. These were evaluated by calculating the percentage area of visible covering and colony counting method, respectively. The secondary outcome were the prevalence of the main investigated risk factors and their influence on the effectiveness of the cleaning protocols. Sample size calculation was based on the primary outcome, presuming a superiority trial with statistical tests performed with 80% power and α=0.05. Considering the data from a previous study,^26^ 15 participants per group would be sufficient to detect statistically significant differences. Nevertheless, considering possible dropouts, 30% were added to each group, thus resulting in a final sample size of n=20.

**Figure 1:**
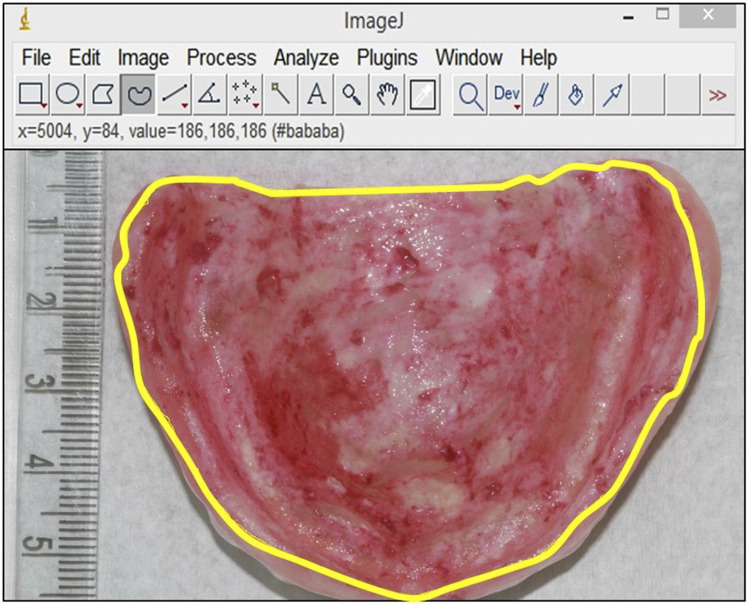
Area of stained biofilm in the internal surface of the MCD delimited (yellow line) by the image processing software (Image J 1.54f)

Before and after the application of the protocols, the biofilm of the internal surface of MCD was shown using a 1% neutral red solution (Sigma-Aldrich Inc., St. Louis, MO, USA), a dye that can efficiently highlight biofilm, that is easily removed, and that has no antimicrobial effect.^20^ The surfaces were then photographed with a digital camera (Canon EOS Rebel T6i, Sony, Tokyo, Japan) fixed on a stand (CS-4, Testrite Inst. Co., Inc., Newark, NJ, USA) under the same conditions (place, light, angle, and operator).^20^ The photographs were then transferred to a computer to measure the total internal surface area and areas corresponding to the stained region using an image processing software (ImageJ 1.54f) (Figure 1). The percentage of the denture biofilm coverage area was calculated by using the ratio between the biofilm area multiplied by 100 and the total surface area of the internal denture base.^13,20^This procedure was performed by a single observer who was blind to the study group and period.

For microbiological quantitative cultures, the collected material was cultured in duplicates before and after the application of the protocols. Microbiological samples were obtained by vigorously rubbing sterile oral swabs on the intaglio surface of the MCD for 1 min.^16^ Each swab was placed in a test tube containing 5 mL of 0.9% sterile saline. Next, to detach the collected material from the swabs, the tubes were placed in a tube holder inside an ultrasonic cleaning tank (Cristófoli, Campo Mourão, PR, Brazil) with cold water (6 to 10ºC) and were ultrasonicated for 20 min. Then, each tube was vigorously vortexed for 1 min before a 10-fold serial dilution. Aliquots (25 μL) of each dilution were seeded in blood agar (New Prov Produtos para Laboratório, Ltda, Pinhais, PR, Brazil) to the main genera of oral and non-oral bacteria (including the most important respiratory pathogens) and in Sabouraud dextrose agar (Sigma-Aldrich Inc., St. Louis, MO, USA) to detect *Candida* spp. The blood agar plates were incubated at 37°C in a capnophilic atmosphere (5% CO_2_) for 24-48 h, and the Sabouraud dextrose agar plates were incubated at 37°C for 48 h. Then, viable colonies were quantified and the colony forming units per milliliter (CFU/mL) were determined. The same operator carried out all these procedures.

To ensure homogeneity between the study groups, the demographic characteristics of participants and their risk factors were statistically analyzed using the Kruskal-Wallis test, one-way ANOVA, and the chi-squared (χ^2^) test. The diagnostic hypotheses indicated in the medical records were grouped into large categories (H1, H2, H3, ...), according to the chapters of the International Statistical Classification of Diseases and Related Health Problems (ICD) (World Health Organization, ICD-11, 2022).^40^ The problems most frequently observed in participants were disorders of the respiratory, circulatory, genitourinary systems, among others. The Kruskal-Wallis test was applied to these data to assess whether a difference between the study groups could be found regarding the diagnostic hypothesis before the different proposed denture cleaning protocols. The same test was applied after the evaluated protocols to assess whether the hypotheses influenced their effectiveness.

Even with the transformation of the numbers of CFU/mL into logarithms, the resulting data distribution followed an abnormal distribution. Also, there was no normality and homogeneity for the percentage values of denture biofilm coverage area. Thus, the Wilcoxon test was used to analyze each denture cleaning protocol before and after its application. The comparison between the groups in the different evaluation periods was performed by using the Kruskal-Wallis test, followed by the Bonferroni post hoc test (α=.05). Pearson’s correlation coefficient was applied to verify whether the quantitative variables (log_10_ CFU/mL values and percentage area of denture biofilm coverage) were correlated. The analyses were performed using two statistical software (SigmaPlot 12.0, Systat Software Inc, Chicago, IL, USA; Statistica 10.0, Dell Inc, Austin, TX, USA).

## Results

**Figure 2:**
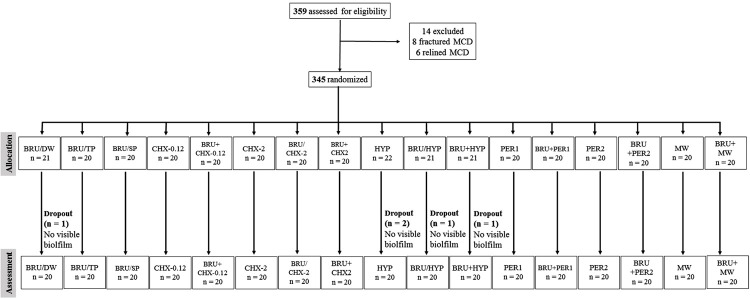
Flow diagram (study groups)

Figure 2 presents the flow diagram of participants and the reasons for exclusions and withdrawals.

No statistically significant differences were observed between study groups for participants’ age and sex (Table 1). Before the denture cleaning protocols, no significant differences were found between risk factors (denture age, nocturnal wear of MCD, and use of drugs) (Table 1). A small but significant difference was observed between groups regarding length of hospitalization (Table 1). These results show that randomization was adequate, making the groups homogeneous regarding demographic characteristics and risk factors.

**Table 1:** Demographic characteristics and risk factor

	Mean age (participant)	% sex		Median time (d) of hospitalization	Median age (MCD)	% drugs			% nocturnal wear of MCD
Groups		M	F			ATB	ATF	STE	
BRU/DW	80.6	45	55	5	10	20	10	10	35
BRU/TP	75.1	45	55	3.5	6	40	5	5	40
BRU/SP	71.6	55	45	4.5	5	30	12	10	35
CHX-0.12	75	45	55	5.5	10	50	5	30	20
BRU+CHX-0.12	75.5	60	40	5	10	35	0	15	20
CHX-2	79.5	75	25	5	10	40	15	20	10
BRU/CHX-2	77	70	30	5	10	45	10	5	15
BRU+CHX-2	73.3	40	60	5.5	10	40	0	5	25
HYP	72.1	35	65	4.5	10	50	10	20	40
BRU+HYP	77	45	55	4	10	55	10	5	20
BRU/HYP	77.9	45	55	2.5	5	50	5	10	20
PER1	77.1	50	50	4.5	10	40	15	15	30
BRU+PER1	73.1	40	60	5.5	7	35	10	15	25
PER2	71.7	30	70	4	10	70	5	10	15
BRU+PER2	73.6	50	50	5	5	30	15	0	25
MW	79.7	65	35	4	10	50	0	10	25
BRU+MW	76.2	65	35	5	7	45	5	30	35
P value	0.453^Ɨ^	0.283*		0.041^#^	0.749^#^	0.892*	0.385*	0.481*	0.506*

Sex: F= female; M= male; Drugs: ATB = antibiotics, ATF = antifungals, STE = steroids. ^Ɨ^ANOVA 1 fator; *χ2; ^#^Kruskal-Wallis (*P*<0.05).BRU/DW: Brushing with distilled water; toothpaste (BRU/TP) or neutral liquid soap (BRU/SP); immersion in cleaning agent only (0.12% chlorhexidine - CLX-0.12, 2% chlorhexidine-CLX-2, 1% sodium hypochlorite-HYP, 1 tablet-PER1 or 2 tablets-PER2 of alkaline peroxide); immersion in agents combined with brushing with water (BRU+CLX0.12; BRU+CLX-2; BRU+HYP; BRU+ PER1; BRU+PER2), 2% chlorhexidine (BRU/CLX-2) or 1% sodium hypochlorite (BRU/HYP), and denture microwaved for 3 min at 650 W only (MW) or combined with brushing with water (BRU+MW).

The most prevalent diagnostic hypotheses in the study groups were diseases of the circulatory system (n=85), followed by diseases of the respiratory system (n=74) (Table 2). Regarding the diagnostic hypotheses for both data from quantitative microbiological cultures (log_10_ CFU/mL values) and those on the percentage area of denture biofilm coverage, the Kruskal-Wallis test demonstrated no difference between the study groups before (*P*=0.213 and *P*=0.281, respectively) and after (*P*=0.327 and *P*=0.060, respectively) the application of denture cleaning protocols. Thus, there was homogeneity of the subsamples in the initial period regarding the diagnostic hypotheses associated with the microbial colonization of dentures and they failed to skew the hygiene protocols evaluated in this study.

**Table 2:** Distribution of diagnostic hypotheses in study groups

Groups	H1	H2	H3	H4	H5	H6	H7
BRU/DW	4	4	1	2	2	5	2
BRU/TP	0	4	2	2	1	6	5
BRU/SP	7	2	1	1	2	6	1
CHX-0.12	7	1	0	1	0	3	8
BRU+CHX-0.12	3	3	2	0	0	9	3
CHX-2	2	4	4	3	0	7	0
BRU/CHX-2	1	1	4	4	2	4	4
BRU+CHX-2	3	8	2	0	0	4	3
HYP	0	6	5	1	2	5	1
BRU+HYP	5	5	0	1	1	3	5
BRU/HYP	1	6	0	1	1	9	2
PER1	2	3	3	1	2	4	5
BRU+PER1	3	4	3	2	1	4	3
PER2	2	4	3	5	2	2	2
BRU+PER2	4	3	3	2	3	4	1
MW	0	6	3	0	0	6	5
BRU+MW	1	10	2	0	0	4	3
Total	45	74	38	26	19	85	53

H1: External causes of morbidity or mortality; H2: Diseases of the respiratory system; H3: Diseases of the genitourinary system; H4: Diseases of the digestive system; H5: Diseases of the skin; H6: Diseases of the circulatory system; H7: Other hypotheses.

**Figure 3:**
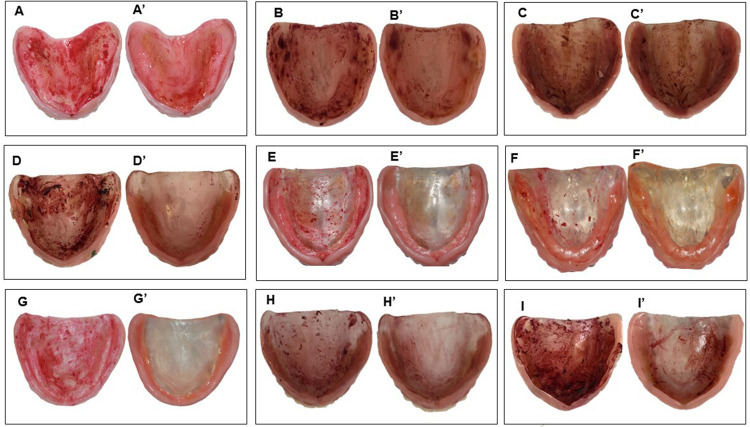
Biofilm disclosed on the internal surface of MCD before (left) and after (right) application of the following cleaning protocols: BRU/DW (A and A’), BRU/SP (B and B’), CHX-0.12 (C and C’), CHX-2 (D and D’), BRU/CHX-2 (E and E’), HYP (F and F’), BRU/HYP (G and G’), BRU+PER2 (H and H’), and MW (I and I’)

Compared with baseline, there was a significant reduction in the median values of the percentage area of denture biofilm after the application of the protocols in all study groups (*P*<0.001) (Table 3). The BRU/HYP group demonstrated the highest percentage reduction in the area of biofilm disclosed on the internal surface of the MCD (*P*<0.05), which was not statistically different from the median values of the BRU/CHX-2, HYP, BRU+HYP, and BRU+MW groups (*P*>0.05), which in turn were similar to those of the BRU+ CHX-2 group (*P*>0.05) (Table 3). The lowest percentage reduction was observed for the control group with distilled water (BRU/DW) (*P*<0.05), which was not statistically different from the control group with toothpaste (BRU/TP) and the following experimental groups: CHX-0.12, BRU+CHX-0.12, PER1, PER2, and MW (*P*>0.05) (Table 3). These results are illustrated in Figure 3. The groups that used 1% sodium hypochlorite in association with brushing (BRU+HYP; BRU/HYP) were not statistically different from the group that used it without brushing (HYP) (*P*>0.05) (Table 3). This finding was consistent with the absence of biofilm observed in more than half of the dentures in these three groups (38/60).

**Table 3:** Medians (interquartile ranges) of the percentage area of denture biofilm coverage in the study groups before and after the application of denture cleaning protocols

	Before		After	
BRU/DW	79.00 (0.58-0.86)	Aa	69.00 (0.53-0.79)	Ab
BRU/TP	66.50 (0.49-0.87)	Aa	71.50 (0.22-0.78)	ABb
BRU/SP	69.00 (0.43-0.75)	Aa	18.75 (0.10-0.42)	BCDb
CHX-0.12	77.79 (0.54-0.84)	Aa	58.04 (0.34-0.67)	ABCb
BRU+CHX-0.12	63.10 (0.47-0.79)	Aa	40.55 (0.31-0.51)	ABCb
CHX-2	73.95 (0.53-0.84)	Aa	39.34 (0.22-0.50)	BCDb
BRU/CHX-2	60.10 (0.45-0.76)	Aa	17.40 (0.00-0.25)	FGb
BRU+CHX-2	72.00 (0.57-0.82)	Aa	0.62 (0.00-0.00)	DEFb
HYP	73.50 (0.35-0.88)	Aa	0.00 (0.00-0.12)	EFGb
BRU+HYP	76.50 (0.69-0.88)	Aa	6.30 (0.00-0.36)	EFGb
BRU/HYP	70.10 (0.67-0.78)	Aa	0.00 (0.00-0.00)	Gb
PER1	69.00 (0.55-0.76)	Aa	44.71 (0.34-0.59)	ABb
BRU+PER1	75.52 (0.68-0.86)	Aa	38.39 (0.26-0.50)	CDEb
PER2	64.83 (0.59-0.74)	Aa	42.08 (0.36-0.58)	ABb
BRU+PER2	66.15 (0.54-0.76)	Aa	32.05 (0.22-0.37)	BCDb
MW	69.41 (0.51-0.80)	Aa	55.24 (0.36-0.68)	ABCb
BRU+MW	77.41 (0.45-0.84)	Aa	0.00 (0.00-0.00)	EFGb

Medians accompanied by different lowercase letters within row were significantly different (Wilcoxon test; *P*<0.05)

Medians connected by different capital letters within columns were significantly different (Bonferroni test; *P*<0.05).

A significant reduction in the microbial load of blood agar cultures was observed after the application of all cleaning protocols (*P*<0.05) (Table 4). A more significant reduction in the median values of log_10_ CFU/mL was found for the groups that used solutions of 2% chlorhexidine and 1% sodium hypochlorite and microwave irradiation (CHX-2, BRU+CHX-2, BRU/CHX-2, HYP, BRU+HYP, BRU/HYP, MW, and BRU+MW) compared with the other study groups (*P*<0.05), which were significantly indifferent (*P*>0.05) (Table 4). The association of brushing with distilled water (BRU+CHX-2, BRU+HYP, and BRU+MW) or chemical agents (BRU/HYP and BRU/CHX-2) resulted in no statistically better effectiveness of the protocols than the methods alone (CHX-2, HYP, and MW) (*P*>0.05; Table 4).

**Table 4:** Values in log10 CFU/ml of the medians (interquartile ranges) of the viable microbial colonies in the study groups before and after the application of denture cleaning protocols

	BLOOD AGAR				SABOURAUD AGAR			
Groups	Before		After		Before		After	
BRU/DW	12.07 (11.62-13.07)	Aa	4.70 (4.07-5.18)	ABb	7.51 (5.67-8.92)	ABCa	5.21 (4.07-5.80)	Ab
BRU/TP	12.00 (11.46-12.41)	Aa	7.99 (5.63-10.07)	Ab	6.47 (5.61-8.75)	ABCDEa	4.84 (4.07-5.31)	ABb
BRU/SP	12.10 (7.90-13.03)	Aa	4.97 (4.07-5.44)	ABb	4.09 (3.45-5.02)	DEa	3.65 (0.00-4.06)	BCDb
CHX-0.12	11.86 (11.51-12.46)	Aa	5.63 (4.09-6.20)	ABb	6.02 (4.47 - 6.34)	BCDEa	0.00 (0.00 - 2.83)	CDEb
BRU+CHX-0.12	11.95 (11.25-12.07)	Aa	2.15 (0.00-4.60)	ABb	8.91 (5.73 - 9.31)	ABCa	0.00 (0.00 - 0.00)	DEb
CHX-2	11.64 (11.27-12.02)	Aa	0.00 (0.00-0.00)	CDb	8.82 (8.28 - 8.93)	ABa	0.00 (0.00 - 0.00)	Eb
BRU/CHX-2	11.82 (11.30-12.30)	Aa	0.00 (0.00-0.00)	Db	6.73 (5.91 - 8.71)	ABCDa	0.00 (0.00 - 0.00)	Eb
BRU+CHX-2	11.78 (11.41-12.35)	Aa	0.00 (0.00-0.00)	Db	4.97 (0.00 - 0.00)	CDEa	0.00 (0.00 - 0.00)	Eb
HYP	11.55 (9.89 - 11.97)	Aa	0.00 (0.00-0.00)	CDb	6.05 (5.47 - 9.05)	ABCDa	0.00 (0.00 - 0.00)	Eb
BRU+HYP	11.53 (10.72-12.18)	Aa	0.00 (0.00-0.00)	Db	9.04 (7.97 - 10.07)	Aa	0.00 (0.00 - 0.00)	DEb
BRU/HYP	12.07 (11.62-13.07)	Aa	0.00 (0.00-0.00)	CDb	8.34 (6.07 - 8.68)	ABCa	0.00 (0.00 - 0.00)	Eb
PER1	11.82 (11.39-12.36)	Aa	4.57 (0.68-6.24)	ABb	6.82 (5.05 - 8.49)	ABCDEa	5.14 (2.30 - 6.39)	ABb
BRU+PER1	12.61 (11.26-13.07)	Aa	4.84 (4.07-5.43)	ABb	5.64 (4.07 - 6.34)	CDEa	4.90 (0.00 - 5.65)	ABb
PER2	11.97 (11.30-12.88)	Aa	5.88 (0.00-8.66)	ABb	5.74 (3.91 - 6.77)	ABCDEa	4.07 (2.40 - 4.31)	ABCb
BRU+PER2	11.55 (9.64-11.99)	Aa	4.70 (0.00-5.98)	BCb	5.35 (4.47 - 6.55)	BCDEa	1.34 (0.00 - 5.35)	BCb
MW	12.09 (11.89-12.59)	Aa	0.00 (0.00-0.00)	CDb	8.60 (5.59 - 7.36)	ABCDa	0.00 (0.00 - 0.00)	DEb
BRU+MW	11.14 (7.890-11.66)	Aa	0.00 (0.00-0.00)	Db	3.45 (2.21 - 4.48)	Ea	0.00 (0.00 - 0.00)	DEb

Medians connected by different capital letters within columns were significantly different (Bonferroni test; *P*<0.05).

Medians accompanied by different lowercase letters within rows were significantly different (Wilcoxon test; *P*<0.05).

Compared with baseline, the numbers of *Candida* colonies in the Sabouraud agar cultures were significantly reduced for all study groups (*P*<0.05) (Table 4). The greatest reduction in fungal load was observed in the Sabouraud agar cultures of the groups that used 1% sodium hypochlorite and 2% chlorhexidine solutions, regardless of the association with brushing (CHX-2, BRU/CHX-2, BRU+CHX2, HYP, BRU/HYP, and BRU+HYP) (*P<*0.05), which were not statistically different from the groups using 0.12% chlorhexidine solution and microwave irradiation with or without brushing (CHX-0.12, BRU+CHX-0.12, MW, and BRU+MW) (*P*>0.05) (Table 4). These last groups were statistically similar to the control group using brushing with soap (BRU/SP) (*P*>0.05), which in turn was indifferent from the other groups (*P*>0.05), except for the control group using water (BRU/DW) (*P<*0.05) (Table 4). This control group showed the smallest numerical reduction in fungal load among all cleaning protocols but was not statistically different from the control group using toothpaste and the following groups of alkaline peroxide: PER1, BRU+PER1, and PER2 (*P*>0.05; Table 4).

No denture was damaged with the cleaning protocols tested in this study. Similarly, there were no complaints from participants when they received their cleaned dentures.

## Discussion

In this study, the main objective was to compare different denture cleaning protocols in hospitalized patients’ MCD to establish an effective protocol for removing denture biofilm, even after a single application. This is justified as the length of stay in hospitals can be relatively short, as observed with the participants in this trial (average of 3.5 to 5.5 days). Therefore, the intention was to define a globalized protocol that could be applicable to complete dentures for all wearers, regardless of their oral condition as this could not be evaluated in relation to improvement within a few days of intervention.

It was necessary to evaluate the effectiveness of the protocols without the interference of demographic characteristics, risk factors, and diagnostic hypotheses (ICD-11). Thus, these aspects were assessed via statistical analysis, and there was homogeneity among the 17 study groups for all risk factors and diagnostic hypotheses. Consequently, their potential contribution to the results obtained with the tested protocols was possibly eliminated. In this trial, 56.2% of participants were women, and their mean age was 71.72 years, corroborating the findings of previous studies.^4,6,13,20,28,36^The median age of MCD was over five years, as previously observed,^6,16^and the nocturnal wear of dentures was present in most (65.5%) individuals in this study. These data are worrying as difficulties in swallowing and the use of complete dentures during sleep resulted in a 2.3 times greater risk of aspiration pneumonia, comparable with the main predisposing factors such as history of stroke, respiratory disease, and cognitive impairment.^6^

According to the results obtained by microbiological cultures and denture biofilm coverage area, the hypothesis was accepted since cleaning protocols differed in relation to their effectiveness. When combining the findings of the biofilm coverage area and the microbiological (blood agar) and mycological (Sabouraud agar) cultures, greater effectiveness was observed among all tested denture cleaning protocols, for those that used immersion in a 1% sodium hypochlorite solution, regardless of the association with brushing (HYP, BRU+HYP, and BRU/HYP), the group of brushing with 2% chlorhexidine solution (BRU/CHX-2), and the group associating brushing with microwave irradiation (BRU+MW).

Sodium hypochlorite is one of the oldest chemical agents used for cleaning and disinfecting complete dentures. Its benefit derives from its fungicidal and bactericidal action and its ability to dissolve organic material, calculus, and mucin.^41^ This capacity may be associated with its alkaline pH (pH>11), which acts in dissolving the cells inserted in the denture biofilm by increasing the electrostatic repulsion between them and the surface of the material.^41^ A disadvantage of sodium hypochlorite solutions is their potential residual cytotoxic effect as these agents are impregnated in the irregularities and porosities of the acrylic resins of denture bases after disinfection.^25^ To minimize this effect, alkaline solutions should be used in lower concentrations, such as the 1% used in this investigation, which failed to increase the slight cytotoxic effect of the acrylic resin on human gingival fibroblasts even after successive cycles of daily overnight immersion (8h), simulating the periods of 9 months or 1.5 year.^25^ This concentration and exposure time were selected based on the antimicrobial effectiveness demonstrated by *in vitro* and *in vivo* studies on denture disinfection after a single cycle of immersion of denture base materials (or complete denture) for 10 min.^17,18,19,23^ Unlike the results obtained in this trial, Pellizzaro, et al.^24^ (2012) reported greater antimicrobial effectiveness when immersion in 1% sodium hypochlorite was associated with brushing acrylic resin specimens. Such differences can be attributed to the fact that the study was carried out under *in vitro* conditions with a biofilm of a single microbial species and to the shorter immersion time than that used in this investigation.

The selection of concentrations, immersion time, and brushing protocols used with chlorhexidine solutions was carried out based on the antimicrobial effectiveness demonstrated by *in vitro*^15,17,18,20,22,24,27^and *in vivo* studies.^23,25^ In this investigation, brushing with 2% chlorhexidine (BRU/CHX-2) proved as effective as protocols that used hypochlorite solution. Brushing with water followed by immersion in 2% chlorhexidine (BRU+CHX-2) showed similar results, with differences in removing denture biofilm only in the hypochlorite brushing group (BRU/HYP). Although chlorhexidine used alone (CHX-2) was as effective as hypochlorite or hypochlorite with brushing in reducing the microbial and fungal load, it resulted in a lower reduction percentage in the biofilm coverage area. The mechanism of the antibacterial action of chlorhexidine is related to its dicationic molecular structure, with rapid attraction to the surface of the bacterial cell, normally negatively charged, which results in a bond that alters the integrity of the cell membrane.^42^Additionally, this antiseptic has substantivity, which ensures its gradual release at the site of use for prolonged periods.^42^ Despite these effects, chlorhexidine solutions lack the same ability to dissolve organic material as hypochlorite, which explains its greater effectiveness in removing denture biofilm when associated with brushing. Chlorhexidine 0.12% (CHX-0.12), regardless of the association with brushing, showed a significant reduction in its ability to reduce the microbial load and the denture biofilm coverage area. This antiseptic has distinct effects at different concentrations, which vary between microbial species, with bactericidal/fungicidal action at higher concentrations^17,18,22^ and bacteriostatic action at low concentrations.^17,27,43^

Denture microwave irradiation showed comparable effectiveness with protocols with sodium hypochlorite in reducing microbial and fungal load. The microwave irradiation protocol selected in this study was previously defined in a clinical study that demonstrated its effectiveness in disinfecting dentures to prevent cross-contamination.^26^ Although the mechanism of antimicrobial action of microwaves is yet unclear, the most accepted theory is that, in addition to the lethal effects resulting from the heat generated during irradiation (thermal), there are effects that evade explanations by this premise alone (non-thermal) because the destruction of microorganisms by microwaves has been observed at temperatures below the point of thermal destruction. Thus, the death of microorganisms probably results from the interaction of the electromagnetic field with the cell molecules and the surrounding liquid medium, creating effects that are not only caused by thermal action.^44^A disadvantage of microwave irradiation in hospitals is the need for at least one oven at each nursing station in the ward, which is unnecessary with denture cleaners that can be used, for example, in the bathroom sink of the patient’s room. Despite the favorable results obtained with microwaves in microbial and fungal cultures, similar to those of 2% chlorhexidine protocols, irradiation of dentures alone demonstrated less effectiveness in removing biofilm than when combined with brushing. In addition to reducing the microbial load, this removal is always intended in denture cleaning protocols since the residual biofilm, composed of non-viable cells, can act as a source of endotoxins, favoring their rapid recolonization and allowing protection of the new pathogens.^45^

The selection of cleaning protocols with alkaline peroxide in this study using one or two tablets was carried out based on previous investigations that showed its *in vitro*^21,39^and *in vivo*^13,14^antimicrobial effectiveness. Alkaline peroxide solutions are known to promote the mechanical removal of debris and stains on dentures by the action of oxidizing agents resulting from the decomposition of sodium perborate and sodium percarbonate.^46^ They are also recommended for controlling denture biofilm,^13,23,47^in addition to the advantages of absence of odor and aftertaste and lower cytotoxicity.^48^ Despite this, in this RCT, regardless of its association with brushing or the use of one or two tablets, peroxide-based solutions showed antimicrobial action similar to that of the control protocols (BRU/DW, BRU/TP, and BRU/SP) both in reducing biofilm coverage area and microbial and fungal load. Similarly, Uludamar, et al.^46^ (2010) reported no differences between these solutions and the control group (water) in reducing the fungal load of the dentures of participants with denture stomatitis. Other clinical studies have also demonstrated the ineffective action of alkaline peroxides against *Candida albicans* on denture biofilm,^13,49,50^ which otherwise showed favorable antibacterial activity.^13,49^

In this investigation, although they reduced the denture biofilm coverage area and the microbial and fungal load, the control groups that used brushing with distilled water, toothpaste, or liquid soap showed significantly lower efficacy than the groups using solutions of 1% sodium hypochlorite and 2% chlorhexidine. Despite being the most used cleaning method by denture users, brushing with water,^12,13,49^ neutral soap,^11,14,16^ and toothpaste^37^ were found to be ineffective for reducing the microbial and/or fungal load of denture biofilm. Similarly, brushing with water was the least effective *in vivo* method for removing denture biofilm coverage^36^, as well for reducing *C. albicans*, mutans *Streptococci*, and other aerobic species in denture biofilm.^13^When inserted into a biofilm, microorganisms become partially protected from the shear forces of the toothbrush, minimizing its action, which is also hampered by irregularities and porosities in the acrylic resin of denture bases.^49^Among the evaluated control protocols, brushing with soap was statistically better than brushing with water in removing visible biofilm and reducing fungal load. The active components present in the tested soap, such as ethylenediaminetetraacetic acid (EDTA), citric acid, sodium hydroxide, curcumin, and cocamidopropyl betaine, may have contributed to the better action of this product, which demonstrated *in vivo* effectiveness in reducing *Candida* spp. in maxillary complete dentures.^28^Thus, the result of this study with liquid soap suggests using this hygiene product in home denture cleaning practices to control biofilm.^38,47^

A limitation of this study was that denture cleaning protocols were performed only once and exclusively on maxillary dentures. Future studies should include the application of protocols throughout the patient’s stay in the hospital, which, in this clinical trial, ranged from 3.5 to 5.5 days, in addition to considering mandibular dentures. Caution should be taken regarding the application of the protocols of this study to other types of removable dentures, such as partial dentures with a metal framework.

Many hospitals, such as the co-participants in this research, are yet to establish oral hygiene protocols or professionals specialized in oral health. Such procedures are relevant not only to ICU patients but to those admitted to the ward, most of which, as the participants in this study, have removable dentures. The clear association between oral and systemic diseases suggests that removable dentures constitute a microbial reservoir with a high concentration of respiratory pathogens.^3-5^ As a result, hygiene protocols aimed at reducing denture biofilm, especially in older individuals and frail hospitalized individuals at greater risk of respiratory infections, must be adopted.^35^ For the effective removal of denture biofilm and reduction of microbial and fungal load, the results of this RCT suggest the adoption of protocols using 1% sodium hypochlorite, regardless of the association with brushing. Simply immersing the dentures in this solution for 10 min, as an effective control protocol for denture biofilm during the patient’s hospitalization, can represent an easy-to-execute, rapid, and affordable method for hospitals, even in comparison with solutions used in the hospital routine such as chlorhexidine digluconate. Additionally, the adoption of a standard and effective protocol such as the one suggested by this study may result in a shorter hospital stay for the patient both by preventing infections associated with denture biofilm and by reducing the course of virulence of a previously established lung disease, leading to lower hospitalization costs.

## Conclusions

All tested denture cleaning protocols resulted in a significant reduction in both the coverage area and the microbial and fungal load of the biofilm on the dentures of hospitalized patients, with the best results observed for those using 1% sodium hypochlorite. A single immersion in this solution for 10 min, even in the absence of brushing, proved to be a practical, straightforward, and affordable option for cleaning the complete dentures of hospitalized patients.
